# Weight loss before conception: effects on atherogenic apolipoprotein lipid particles and endothelial function during pregnancy

**DOI:** 10.1016/j.xfre.2025.02.009

**Published:** 2025-02-26

**Authors:** Robert A. Wild, Daniel Zhao, Michael J. McPhaul, Rodney K. Edwards, Karl R. Hansen, Elizabeth L. Wolfe, David S. Wrenn

**Affiliations:** a Department of Obstetrics and Gynecology, University of Oklahoma Health Sciences Center, Oklahoma City, Oklahoma; b Biostatistics and Clinical Epidemiology, University of Oklahoma Health Sciences Center, Oklahoma City, Oklahoma; c Quest Diagnostics Nichols Institute, San Juan Capistrano, California

**Keywords:** LDL-cholesterol particles, ADMA, obesity, pregnancy, weight loss

## Abstract

**Objective:**

To assess atherogenic apolipoprotein lipids and markers of vascular function in pregnancy after prepregnancy weight loss.

**Design:**

Retrospective cohort study.

**Subjects:**

The weight loss cohort included pregnant women who achieved prepregnancy weight loss, and the weight gain cohort consisted of those who gained weight before pregnancy. All patients became pregnant after enrolling in the randomized clinical trial “Improving Reproductive Fitness Through Pretreatment with Lifestyle Modification in Obese Women with Unexplained Infertility” and delivered a single live birth at ≥36 weeks of gestation.

**Exposure:**

Prepregnancy weight loss.

**Main Outcome Measures:**

Apolipoprotein lipid levels.

**Results:**

Prepregnancy weight loss was associated with lower atherogenic apolipoprotein lipid levels during pregnancy and better indicators of vascular function.

**Conclusion:**

Our findings suggest that encouraging prepregnancy weight loss in obese women leads to improved vascular function during pregnancy. Prepregnancy weight loss has significant implications for healthcare professionals because it underscores the potential benefits of weight loss interventions in reducing the risk of cardiovascular disease for women who become pregnant.

In pregnancy, obese women are at increased risk of preeclampsia, a condition associated with cardiac disease later in life. Biomarkers, including small dense low-density lipoprotein cholesterol (sdLDL-C) particles and asymmetric dimethylarginine (ADMA), are involved in the development of both these conditions (1, 2) (Fig. 1). Small dense low-density lipoprotein cholesterol is considered one of the most atherogenic. When protective vascular nitric oxide (NO) production is compromised, the ADMA level increases, reflecting vascular dysfunction. Weight loss is recommended for obese adults to reduce the risk of cardiovascular disease, and these biomarkers are followed clinically. Although weight loss also reduces the risk of preeclampsia (3), very little is known about how prepregnancy weight loss impacts these biomarkers in pregnancy. Our objective was to measure clinically relevant biomarkers, including highly atherogenic apolipoprotein lipid particles and ADMA, in obese pregnant women who participated in a prepregnancy weight loss intervention.

**Figure 1:**
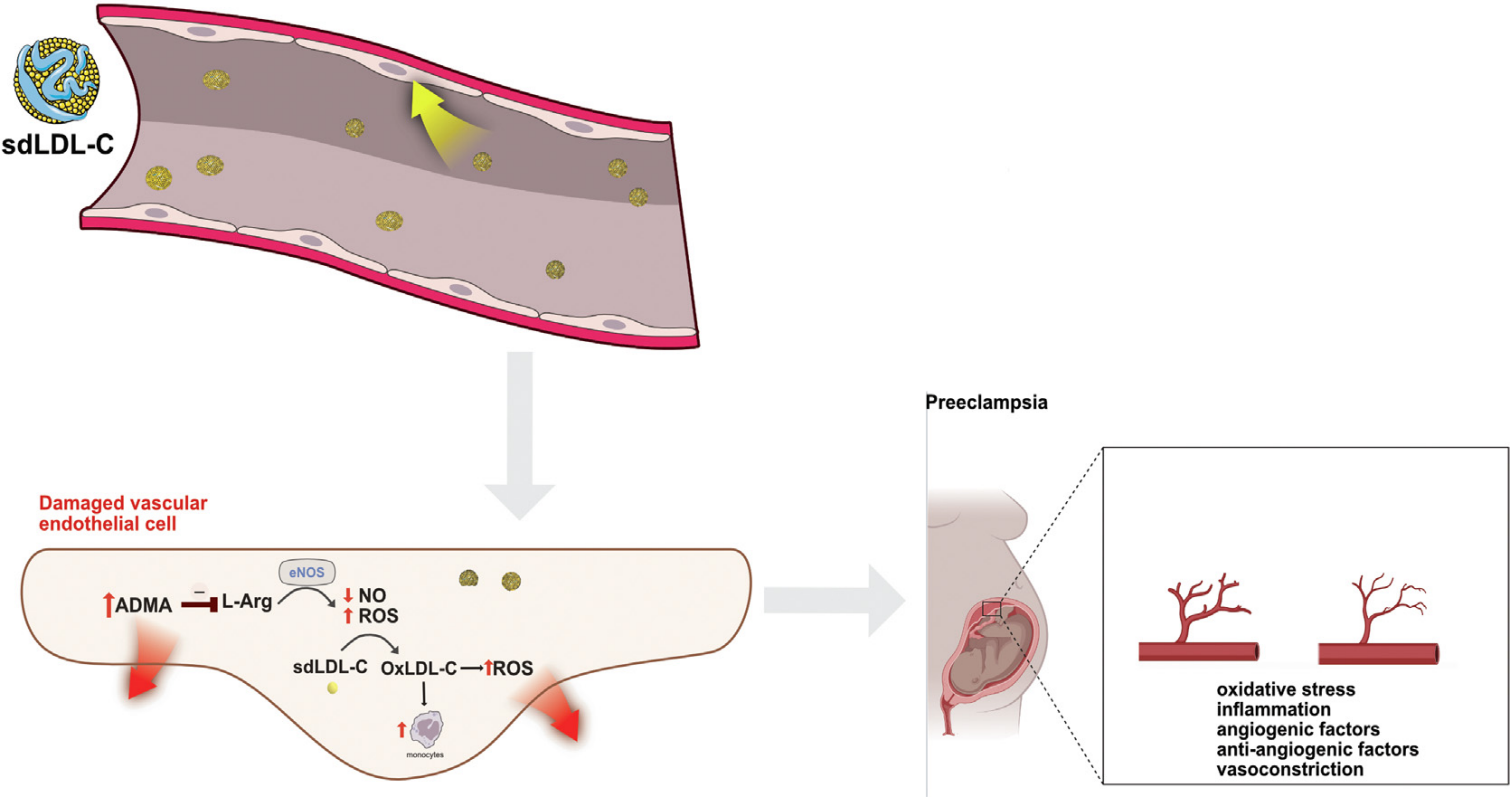
Atherogenic small dense low-density lipoprotein (sdLDL) particles, colloquially “bad cholesterol,” infiltrated the vascular endothelium, where oxidative modification by reactive oxygen species (ROS) triggered inflammation and oxidative stress. This process promoted monocyte recruitment, cytokine release, and endothelial dysfunction by uncoupling endothelial nitric oxide (NO) synthase, producing superoxide anion (O2–) instead of NO, depleting NO bioavailability. Asymmetric dimethylarginine (ADMA) further exacerbated NO depletion by antagonizing L-arginine, the substrate for NO synthase. The combined effects of sdLDL, ROS, and ADMA resulted in vasoconstriction and impaired endothelial function. In preeclampsia, the levels of ADMA and LDL-C particles increased, and the placenta was vulnerable to oxidative stress and inflammation, causing vasoconstriction and ultimately leading to impaired spiral artery remodeling, placental hypoxia, and ischemia. Reducing these particles with weight loss before pregnancy could lower the particle levels and potentially improve vascular dysfunction.

## Materials and methods

### Study design

#### Secondary analysis of the Improving Reproductive Fitness Through Pretreatment with Lifestyle Modification in Obese Women with Unexplained Infertility trial

We evaluated pregnant patients who delivered a singleton live birth within the clinical trial Improving Reproductive Fitness Through Pretreatment with Lifestyle Modification in Obese Women with Unexplained Infertility (FIT-PLESE) (NCT 02432209). The FIT-PLESE trial was conducted nationwide at 9 academic health centers from 2015–2019 (4). In the FIT-PLESE trial, 379 women with obesity (defined as a body mass index [BMI] of ≥30 kg/m^2^) and unexplained infertility were randomly assigned in a 1:1 ratio to 1 of 2 preconception lifestyle modification regimens immediately before infertility treatment with clomiphene citrate and intrauterine insemination. The intensive group underwent increased physical activity and weight loss (target of 7%) through meal replacements and medication (Orlistat) compared with a standard group with increased physical activity alone without meal replacement and weight loss medication. After 16 weeks, participants proceeded to ovarian stimulation (clomiphene citrate) and intrauterine insemination fertility treatment. Fasting blood samples were obtained before and after 16 weeks of lifestyle intervention before conception and at 16, 24, and 32 weeks of pregnancy for those who successfully conceived. All women in this study delivered singleton live births at ≥36 weeks of gestation.

### Biomarkers

• Small dense low-density lipoprotein cholesterol: an atherogenic apolipoprotein lipid particle that mediates atherosclerotic cardiovascular disease (ASCVD) and is elevated in preeclampsia.
• Very small low-density lipoprotein cholesterol (LDL-C) particles “a, b, and c”: particles that can enter the vascular endothelium, creating an inflammatory response by releasing vascular adhesion molecules and monocytes (3, 5, 6).
• Asymmetric dimethylarginine: a biomarker of endothelial dysfunction. When elevated, it reflects poor NO production of the vessel wall. It is increased in preeclampsia (7, 8, 9, 10). The circulating ADMA levels naturally decrease with pregnancy, reflecting increased NO production and adaptive vascular blood flow to sustain oxygen delivery to a rapidly growing fetus. Atherogenic cholesterol particles entering the vascular endothelial can disturb NO production and ADMA.

We measured apolipoprotein lipid particles and ADMA levels throughout pregnancy to determine the effects of preconception weight change. The weight loss cohort included pregnant patients in the singleton live birth cohort in the FIT-PLESE trial (4) who successfully lost weight before conception. The weight gain cohort included pregnant patients in the singleton live birth cohort who gained weight before they became pregnant. One person did not change weight; however, she significantly increased her waist circumference and was added to the no-weight-loss cohort.

### Biochemical methods

Ion mobility was used to assess apolipoprotein fractions at Quest Diagnostics Nichols Institute (San Juan Capistrano, CA) (11). Cleveland Heart Lab (Cleveland, OH) assessed ADMA, which was quantified by precipitation with methanol, followed by analysis by liquid chromatography-tandem mass spectrometry using multiple reaction monitoring. Briefly, d6-ADMA (Medical Isotopes, Pelham, NH) internal standards were added to serum samples and were treated with methanol to disrupt hydrophobic binding interactions. The supernatant was removed, diluted, and injected into the liquid chromatography-tandem mass spectrometry system. Separated analytes of interest by normal-phase chromatography on a silica high-performance liquid chromatography column (Atlantis HILIC Silica column, 3 μm, 2.1 × 50 mm) (Waters, Milford, MA) on up to 4 high-performance liquid chromatography systems directly coupled to an Agilent 6494 triple quadrupole mass spectrometer equipped with an Agilent Jet Stream electrospray ionization source in positive ion mode using multiple reaction monitoring and StreamSelect (Agilent, Santa Clara, CA). Calibration was by weighted (1/x) linear regression of the ratio of the peak area of ADMA to that of their respective internal standards. The laboratory used calibration standards of 20–2,000 ng/mL to generate calibration curves to calculate ADMA levels for unknown samples. With additional up to 128-fold dilution, the clinical reportable range extended from 20–256,000 ng/mL for ADMA with interassay imprecision of <2.4%, and extraction recovery for ADMA ranged from 93.4%–105.9%.

### Statistical analysis

The Χ^2^ test, Fisher exact test, and Student *t*-test (or nonparametric alternatives) were used to compare categorical and continuous variables. Skewed values were log-normalized for comparisons. We used general linear models to compare biomarkers by prepregnancy weight change, visit, and interaction. Age was associated with some of the apolipoproteins; therefore, we retained age in the adjusted models. Smoking and treatment arms were not significantly different between the groups and, thus, not included. The Spearman correlations associated ADMA levels with apolipoprotein lipids within weight change categories. We used multiple regression and mediation analysis to associate LDL-C particles with ADMA levels. We used SAS 9.4 (Cary, NC) and NCSS version 2022 (Salt Lake City, UT) for analysis and creating figures.

The institutional review board approved this investigation at the University of Oklahoma Health Sciences Center (institutional review board #10748). The serum was frozen and shipped to the FIT-PLESE central laboratory in Virginia. Frozen serum samples were shipped from Virginia to the Quest laboratories for blinded analysis for this study. A coded identification matched clinical data to the laboratory results. The Quest laboratory director and the statistician were blinded to clinical data. The criteria for inclusion were if the participant had a live birth and if serum for apolipoprotein lipid and biomarker measurements was available.

## Results

Apolipoprotein lipids were measured in 44 patients in the weight loss cohort and 17 in the weight gain cohort. Serum samples were also available to measure ADMA in 18 participants in the weight loss cohort and 13 in the weight gain cohort.

Table 1 shows the clinical variables of both cohorts. The demographics were similar except for weight loss or gain during the 16-week preconception intervention. The mean baseline BMI before any intervention was 39 kg/m^2^. The weight loss cohort had a mean weight loss of −2.7 kg (95% confidence interval [CI], −2.9 to −2.4). The weight gain cohort had a mean weight gain of 1.3 kg (95% CI, 0.8–1.8; *P* <.001). There were almost 6 times more participants in the weight gain cohort who developed preeclampsia than in those who lost weight. Women who successfully lost weight had a longer infertility duration before entering into the study. To check for potential inclusion bias, we compared the clinical variables of participants who delivered live births with unavailable serum (n = 12) with those of participants with available serum. Of these, 67% lost weight, and 33% did not before conception. Their mean age was 32 years. Their BMI averaged 41 kg/m^2^.

**Table 1:** Clinical variables.

Patient characteristics	Weight loss cohort (n = 44)	Weight gain cohort (n = 17)	*P* value
Duration of infertility (mo) (mean, range)	32 (12–132)	18 (6–73)	.002
Age (y) (mean, SD)	31 (± 4)	33 (± 4)	.278
Baseline BMI (kg/m^2^) (mean, SD)	39 (± 6.4)	38 (± 6.3)	.733
Weight change (kg) (mean, (95% CI)	−2.7 (−2.9 to −2.4)	1.3 (0.8–1.8)	<.001
Waist change (cm) (mean, 95% CI)	−6.1 (−7.0 to −5.2)	2.2 (0.78–3.80)	<.001
Race			.342
Hispanic/Latino	9%	17%	
White	71%	63%	
Black	18%	14%	
Asian/Indian	2%	6%	
Smoking			.313
Current	3%	1%	
Former	8%	7%	
Never	40%	14%	
Other/unknown	49%	78%	
Alcohol (current)			.077
Yes	46%	16%	
No	5%	6%	
Unknown	49%	78%	
Education			.643
High school	3%	1%	
Some college	41%	16%	
Graduate school	7%	5%	
Unknown	49%	78%	
Preeclampsia	3/44 (7%)	7/17 (41%)	.005

BMI = body mass index; CI = confidence interval; SD = standard deviation.

**Figure 2:**
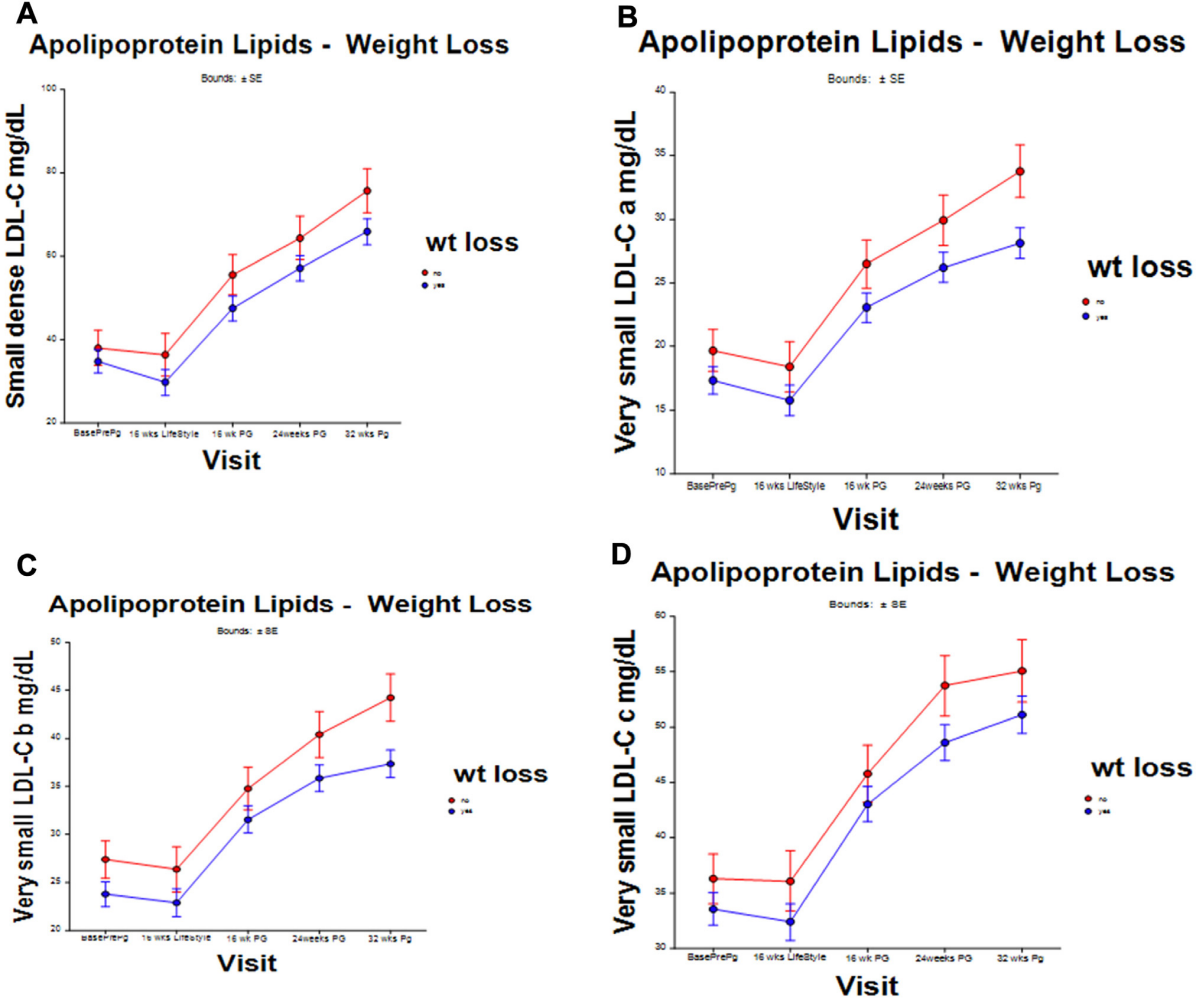
Levels of small dense low-density lipoprotein cholesterol (LDL-C) particles and very small LDL-C particles “a,” “b,” and “c.” Improving Reproductive Fitness Through Pretreatment with Lifestyle Modification in Obese Women with Unexplained Infertility trial: The weight (wt) loss cohort included pregnant participants who lost weight (n = 44) before conception and is in blue. The weight gain cohort is in red—those who did not lose weight and gained (n = 17) before conception.

Figure 2A to D shows the mean age-adjusted sdLDL-C and very small LDL-C particles (a, b, and c) before and after the 16-week preconception weight changes and during pregnancy visits for both cohorts. The levels of these atherogenic apolipoprotein lipids increased in pregnancy in those who gained weight before pregnancy, and differences increased as the pregnancies progressed. Tests for interaction between the weight change groups and the visit were not significant. The mean difference in the sdLDL-C levels during pregnancy between the weight loss and weight gain cohorts was 8 mg/dL (15%) lower (95% CI, 0.37–16.5; *P*=.040) in those who successfully lost weight. At 32 weeks specifically, the mean sdLDL-C was 10 mg/dL lower in the weight loss cohort. The mean values of subparticles “a,” “b,” and “c” were also lower at each visit during pregnancy in those who lost weight before conception. The value of particle “a” was 4.0 mg/dL (16%) lower (95% CI, 1.3–7.3; *P*≤.01). At 32 weeks of pregnancy, the value of particle “a” was 6 mg/dL lower (*P*=.810). For subparticle “b,” the adjusted mean was 5 mg/dL (14%) lower (95% CI, 1.4–8.4; *P*<.01). At 32 weeks, it was 7 mg/dL lower. The value of very small LDL-C “c” was 4 mg/dL (10%) lower (95% CI, 1.2–6.7; *P*=.04). The largest difference for subparticle “c” was at 24 weeks of pregnancy (5 mg/dL). The differences were Bonferroni corrected. The other standard lipids (total cholesterol, LDL-C, high-density lipoprotein cholesterol, and apolipoprotein B levels) were not statistically different between weight change groups during any of the visits (data not shown).

**Figure 3:**
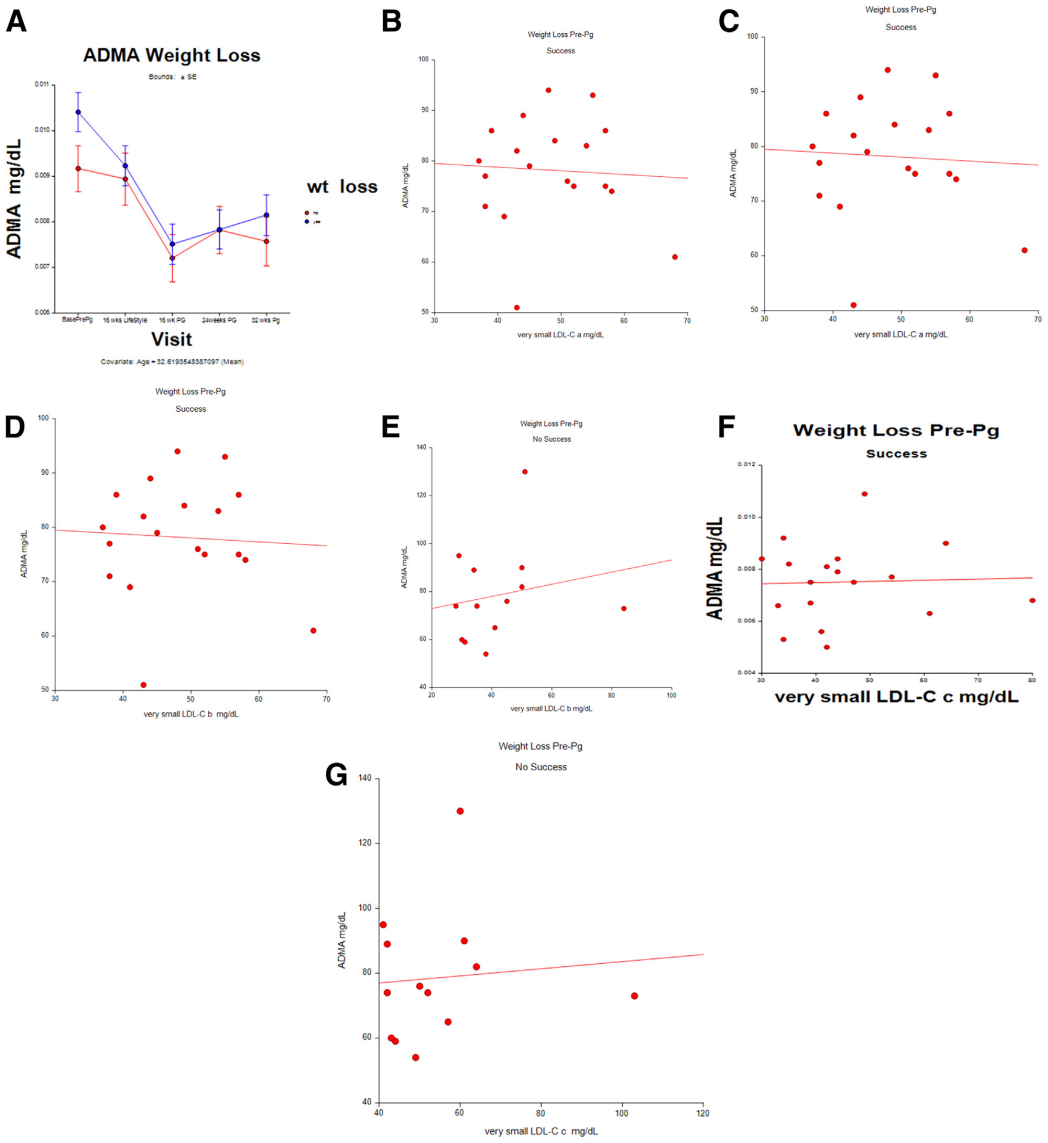
Asymmetric dimethylarginine (ADMA) levels before and during pregnancy (**A**) stratified by pregnant patients who lost weight before conception (blue, n = 18) or did not (red, n = 13)—correlations of low-density lipoprotein cholesterol (LDL-C) particles with ADMA in unsuccessful (**B**) vs. successful (**C**) participants who lost weight.

Figure 3A shows the ADMA levels before conception and during pregnancy. The lowest levels were at 16 weeks and then increased as the pregnancies progressed.

Figure 3B to G shows correlations between atherogenic apolipoprotein sdLDL-C ^‘^a,^’^^‘^b,^’^ and ^‘^c^’^ particles and ADMA levels. The atherogenic particle levels were correlated with ADMA only in those who did not lose weight before conception: small LDL-C ^‘^a,^’^ r = 0.365 (*P*=.04); ^‘^b^’^ r = 0.425 (*P*=.023); and ^‘^c,^’^ r = 0.371 (*P*=.04).

At 16 weeks of gestation, the sdLDL-C particle levels were associated with the ADMA levels when adjusted for absolute weight change before pregnancy (b = 0.003, *P*=.05). In mediation analysis, log-normal sdLDL-C direct effect was beta = 0.00298 (*P*=.05), and the absolute prepregnancy weight change indirect effect was beta = 0.0003479 (*P*=.4332). There was no association between particles and ADMA among those who lost weight before conception.

## Discussion

Patients with infertility with obesity who were pregnant in the FIT-PLESE trial and who successfully lost weight before they conceived had lower circulating levels of highly atherogenic sdLDL-C and lower very small LDL class B subparticle levels during their pregnancies. If participants gained weight before conception, the profiles were more unfavorable. Differences between those who successfully lost weight and those who did not lose and gained weight were even more pronounced as their pregnancies progressed, suggesting that prepregnancy weight loss was associated with better apolipoprotein lipid metabolism throughout the pregnancy. We have shown previously that highly atherogenic particles are associated with preeclampsia and that immediate weight loss lessens the risk for preeclampsia (1, 3). In this analysis, those with better atherogenic, very small LDL-C particles and better endothelial function after weight loss were much less likely to be diagnosed with preeclampsia. Only in those who did not lose weight before conception were the highly atherogenic particle levels correlated significantly with ADMA levels during pregnancy. The particles were not associated with ADMA in people who lost weight. Mediation analysis suggested that sdLDL-C particle excess could be related to ADMA in the women who gained weight, suggesting that these particles could be related to disturbed vascular NO production. Figure 1 illustrates the proposed pathophysiology.

During uncomplicated pregnancies, uterine artery adaptation leads to higher NO levels (8). Expression of NO synthases increases primarily in syncytiotrophoblasts and becomes more active throughout pregnancy, peaking around midgestation (12). Nitric oxide contributes to the vasodilatory action of blood vessels, and physiological blood pressure reduction during pregnancy may greatly rely on this action. Vascular resistance decreases during early pregnancy, when maternal blood volume expands, whereas systemic vascular resistance and systemic blood pressure decline (13). Diminished NO synthesis has been reported previously in pregnancies complicated by preeclampsia (14, 15). Asymmetric dimethylarginine is associated with endothelial dysfunction in uterine artery blood flow disorders (16). Asymmetric dimethylarginine, a natural endogenous and competitive NO synthase inhibitor, is positively correlated with the severity of endothelial dysfunction.

We suggest that the atherogenic particle excess is related to vascular changes and integral to developing preeclampsia. If these increased levels of sdLDL particles contribute to endothelial dysfunction, this is consistent with the Berneis kinetic model during high triglyceride availability (16). In this state, substantial, triglyceride-rich very-low-density lipoprotein particles are reduced to a high level of atherogenic, LDL-C particles with longer plasma half-time because of augmented hepatic lipase hydrolyzation, resulting in more circulating small LDL particles derived from triglyceride-rich very-low-density lipoprotein particles. Vascular endothelial cells are in constant contact with the small and very small LDL-C particles. Endothelial cells metabolize these particles, and endothelial function becomes compromised when overburdened with intracellular cholesterol (16, 17). Direct effects of small atherogenic particles on vascular endothelial cells produce and release diverse signaling molecules that orchestrate vascular physiology by regulating hemostasis, vascular tone, permeability, inflammation, and angiogenesis (17, 18). Dysregulation of NO signaling pathways is fundamental to vascular endothelial damage (19, 20, 21). Very small LDL-C particles have similar inflammatory-inducing properties as sdLDL-C. Increased sdLDL-C particle excess is an independent risk factor for ASCVD (22). If a woman develops preeclampsia, residual structural vascular endothelial damage (23, 24, 25) leads to early ASCVD (23, 26, 27). Further investigation is warranted to understand better the effects of atherogenic dyslipidemias on pregnancy complications and the subsequent risk of ASCVD (28, 29).

Multiple investigations using many study designs are necessary to sort out and amplify some of the hypotheses raised in this secondary analysis. Nutrition intervention studies are complex. Often, there are more changes than a single intervention of interest. Feasibility and inference are consequently limited for small interventional studies. Extensive clinical trial studies are expensive, complex, and often plagued with dropouts, crossovers, or cointerventions. Multiple study designs can provide important information. Comprehensive database epidemiologic studies do suggest that prepregnancy BMI is associated with a higher risk of preeclampsia and small-for-gestational-age infants (30). Preconception and interpregnancy weight gain is associated with an increased risk of gestational diabetes, hypertensive disorders, and preeclampsia during pregnancy (31). The FIT-PLESE cohort is unique because all obese women were electively seeking infertility treatment and were amenable to a weight loss trial. This secondary analysis allowed us to capture the effects of immediate prepregnancy weight change. Most women with obesity who conceive likely do not seek out their physicians to lose weight before conception electively. Therefore, studies of elective weight loss before conception in noninfertility patients are sparse to nonexistent. Cohort studies of pregnant women participating in prenatal research to track neonatal outcomes can be a potential resource (a CHARM study investigation is currently being submitted).

This study has important strengths and limitations. Vigorous blinded methods were applied in the FIT-PLESE trial and this study. Uniquely, before conception, we could study sample participants in each weight change group. We were limited by the serum available to analyze in those who successfully conceived and carried to term delivery. It is reassuring that the clinical variables for the patients without available serum appeared similar to those where we analyzed available serum. The number of persons with serum still available to measure ADMA levels at each pregnancy visit is smaller. Further exploration of mechanistic understanding of how weight loss before conception reduces pregnancy complications is warranted.

## Conclusion

Atherogenic LDL-C particle levels were higher during pregnancy in obese patients who gained weight before conception. In pregnancy, their higher levels of circulating atherogenic, very small LDL-C particles were correlated with ADMA, a biomarker of vascular dysfunction. For those who lost weight, atherogenic particle levels were lower during pregnancy and were not correlated with ADMA. Higher levels of circulating highly atherogenic LDL-C particles are likely detrimental to the vascular system during pregnancy. Weight loss immediately before conception lowers these atherogenic particles throughout their subsequent pregnancy, and this could lead to low risks of preeclampsia and maternal vascular disease over time.

## CRediT Authorship Contribution Statement

**Robert A. Wild:** Writing – review & editing, Writing – original draft, Visualization, Project administration, Methodology, Investigation, Conceptualization. **Daniel Zhao:** Investigation, Formal analysis, Data curation. **Michael J. McPhaul:** Writing – review & editing. **Rodney K. Edwards:** Writing – review & editing. **Karl R. Hansen:** Writing – review & editing. **Elizabeth L. Wolfe:** Writing – review & editing, Resources, Project administration. **David S. Wrenn:** Writing – review & editing.

## Declaration of Interests

R.A.W. reports funding from NIH R03HD101893 (Robert A. Wild, PI—payment to institution) and NIH U10HD077680 (Karl R. Hansen, PI for Reproductive Medicine Network grant—payment to institution) for the submitted work and consulting fees from Ablacare/May Health and Quest Diagnostics outside the submitted work. D.Z. reports that statistical analysis was partially supported by the National Institutes of Health, National Institute of General Medical Sciences (grant 2U54GM104938, PI Judith James), for the submitted work and consulting fees from Tigermed, Inc., outside the submitted work. M.J.M. reports consulting fees from Quest Diagnostics outside the submitted work. R.K.E. reports funding from NIH R03HD101893 (Robert A. Wild, PI—payment to institution) for the submitted work; funding from NIH R01HD113755-01A1 (S. Pierce, PI—payment to institution—topic not related to this article), NIH R01HL120338 (A. Tita, overall PI; R.K.E., site PI—payment to institution), NIH U54GM104938 via OSCTR (J. James, overall PI; P. Parikh project PI—payment to institution), NIH R01HD101476 (C. Scifres, overall PI; R.K.E., site PI—payment to institution—topic not related to this article), Oklahoma State DOH (S. Pierce, PI—payment to institution—topic not related to this article), Presbyterian Health Foundation (local grant, co-PI—payment to institution—topic not related to this article), and Harold Hamm Diabetes Center Local grant (co-PI—payment to institution—topic not related to this article) outside the submitted work. K.R.H. reports funding from NIH R03HD101893 (R.A.W., PI—payment to institution) and NIH U10HD077680 (K.R.H., PI for Reproductive Medicine Network grant—payment to institution) for the submitted work, May Health Device trial contract unrelated to work, consulting fees from May Health, and travel support from the American Society for Reproductive Medicine outside the submitted work. E.L.W. has nothing to disclose. D.S.W. is Laboratory Director at Quest Diagnostics.
